# Automatic Path-Planning Techniques for Minimally Invasive Stereotactic Neurosurgical Procedures—A Systematic Review

**DOI:** 10.3390/s24165238

**Published:** 2024-08-13

**Authors:** Reza Monfaredi, Alondra Concepcion-Gonzalez, Jose Acosta Julbe, Elizabeth Fischer, Gabriel Hernandez-Herrera, Kevin Cleary, Chima Oluigbo

**Affiliations:** 1 Sheikh Zayed Institute of Pediatrics Surgical Innovation, Children’s National Hospital, Washington, DC 20010, USA; emfischer2@childrensnational.org (E.F.); kcleary@childrensnational.org (K.C.); 2 Department of Pediatrics and Radiology, George Washington University, Washington, DC 20037, USA; 3 School of Medicine and Health Sciences, George Washington University School of Medicine, Washington, DC 20052, USA; aconcepcion@gwmail.gwu.edu; 4 Department of Orthopaedic Surgery & Orthopaedic and Arthritis Center for Outcomes Research, Brigham and Women’s Hospital, Boston, MA 02115, USA; jose.acosta14@upr.edu; 5 Mayo Clinic, College of Medicine and Science, Rochester, MN 55905, USA; hernandez-herrera.gabriel@mayo.edu; 6 Department of Neurology and Pediatrics, George Washington University School of Medicine, Washington, DC 20052, USA

**Keywords:** trajectory, path, planning, brain, neurosurgery, automatic

## Abstract

This review systematically examines the recent research from the past decade on diverse path-planning algorithms tailored for stereotactic neurosurgery applications. Our comprehensive investigation involved a thorough search of scholarly papers from Google Scholar, PubMed, IEEE Xplore, and Scopus, utilizing stringent inclusion and exclusion criteria. The screening and selection process was meticulously conducted by a multidisciplinary team comprising three medical students, robotic experts with specialized knowledge in path-planning techniques and medical robotics, and a board-certified neurosurgeon. Each selected paper was reviewed in detail, and the findings were synthesized and reported in this review. The paper is organized around three different types of intervention tools: straight needles, steerable needles, and concentric tube robots. We provide an in-depth analysis of various path-planning algorithms applicable to both single and multi-target scenarios. Multi-target planning techniques are only discussed for straight tools as there is no published work on multi-target planning for steerable needles and concentric tube robots. Additionally, we discuss the imaging modalities employed, the critical anatomical structures considered during path planning, and the current status of research regarding its translation to clinical human studies. To the best of our knowledge and as a conclusion from this systematic review, this is the first review paper published in the last decade that reports various path-planning techniques for different types of tools for minimally invasive neurosurgical applications. Furthermore, this review outlines future trends and identifies existing technology gaps within the field. By highlighting these aspects, we aim to provide a comprehensive overview that can guide future research and development in path planning for stereotactic neurosurgery, ultimately contributing to the advancement of safer and more effective neurosurgical procedures.

## 1. Introduction

### 1.1. Motivation and Scope

Currently, the path planning for stereotactic neurosurgical applications is primarily manual. Manual path planning for a straight tool involves selecting two points to define the optimal needle insertion path based on the surgeon’s experience, anatomical knowledge, and imaging-based visualization of the target tissue or organ.

Surgeons use different imaging modalities, such as magnetic resonance imaging (MRI), computed tomography (CT), or ultrasonography (US), to plan the surgical path with a reasonable safety margin from critical structures such as vessels and identify a feasible and optimal path for the surgical instrument to reach the target site. Optimization criteria, for example, include maximum penetration to the target region, shortest needle length, minimum penetration to white matter, and maximum penetration to gray matter.

Currently in this process, the physician manually selects the entry point on the body surface and a target point inside the body to define the trajectory of the needle based on their judgment and experience. While manual path planning can be effective in some cases, it is a time-consuming and complex process that requires significant expertise and knowledge. Therefore, in recent years, there has been increasing interest in using automatic or semi-automatic computational path planning, which can streamline the process and potentially improve the accuracy and safety of neurosurgical procedures. 

Computational path planning uses algorithms to automatically generate an optimal path to the target based on the patient’s anatomy. This method can benefit procedures requiring complex approaches or multiple instrument placements. However, these algorithms also come with certain limitations and challenges. For instance, it is challenging to estimate needle deflection as it enters through different layers of tissue. In addition, intra-operative replanning may be important in neurosurgery since puncturing the skull can result in brain shift [1]. All these considerations make manual path planning more challenging and time intensive. Many of these challenges can be automatically addressed and compensation techniques can be established using computational path-planning techniques.

Different path-planning algorithms initially developed for mobile robots and manipulators could be used to develop a path planner for minimally invasive procedures. However, it should be noted that the start and the goal points are given in the context of path planning for mobile robots and manipulators. The path planner outputs a safe path from the start point to the target point given the coordinates of the start and goal points. However, unlike path planning in robotics for which the path planning is performed based on a known starting point and goal point, in neurosurgical procedures and in percutaneous procedures in general, the start points (entry point) and even the goal point initially should be planned. Once the start and the goal points are known, then the start point could be connected to the goal point through a straight trajectory for straight tool or a curvilinear trajectory could be planned for curved tools using a separate path planner. In the context of an automatic path planner, the entry point is usually selected automatically but the goal point (target point) can be defined either by a neurosurgeon or automatically selected based on the clinical application type. Using a straight tool with a straight trajectory is the current practice. However, different research groups are investigating using curved tools for better access. 

Path planning for different anatomical regions requires avoiding nearby critical structures and introduces different types of challenges such as local respiratory motion, intra-operative path adjustment due to organ shift, and needle deformation due to puncturing multi-layer, multi-stiffness media. Therefore, different intervention tool types require certain path-planning algorithms. For instance, certain types of sampling-based techniques might not be appropriate for path planning of straight-needle-based intervention. 

Biopsy, a Greek-derived word (bio—life; opsi—to see) loosely translated as “view of the living,” is defined as the removal of tissue from living organisms for microscopic examination and diagnosis. The term “Biopsy” was coined by Ernest Besnier into medical terminology in 1879 [2]. Biopsy is often the definitive procedure that provides tissue for microscopic analysis when additional information is required to guide any indicated therapy. It was one of the first percutaneous procedures to be introduced. Since 1883, advances in imaging technology and improvements in cytopathology have contributed significantly towards the development of various percutaneous procedures. With improved visualization techniques, targets can be approached with a greater margin of safety and accuracy. Smaller tissue samples and hence smaller needles are being used for biopsies due to improved cytopathology [3]. Other image-guided percutaneous procedures that evolved from this technological advancement include facet joint injections, arthrography, tumor ablation, and deep brain stimulation (DBS). Facet joint syndrome is treated with a local anesthetic injected into joints along the sides of the vertebrae to alleviate pain. Arthrography is an imaging technique to view joints such as the knee, shoulder, or hip by injecting a contrast medium usually done under fluoroscopy or MRI. Thermal energy is used to treat tumors in the liver, kidney, bone, and lung using a minimally invasive procedure called tumor ablation. DBS is a neurosurgical procedure that is performed for the treatment of several neurological disorders such as essential tremor, Parkinson’s disease (PD), dystonia, obsessive-compulsive disorder (OCD), and epilepsy. In this procedure, electrical impulses are passed through electrodes implanted at specific targets in the brain. 

Various computational path-planning techniques have been modified for use in medical applications. Different methods have been used in percutaneous procedures including conventional Dijkstra’s or A*-based graph search algorithms. Graph-based planning involves representing the planning region by a graph and then finding the shortest path between the target and entry point using graph search algorithms. Other path-planning techniques include random sampling-based search algorithms. Artificial Potential Field (APF) method where a repulsion model [4] is used to keep the trajectory away from the critical structures, Adaptive Hermite Fractal Tree (AHFT), reinforcement learning-based approach [5], risk mapping methods, and various optimization-based plannings. 

Sampling-based planning involves randomly sampling the planning region (the space around and between the target and the entry points), generating a path or set of candidate paths, and proposing it to the neurosurgeon. The most frequently used sampling-based planning methods include Probabilistic Roadmap (PRM), Rapidly Exploring Random Tree (RRT), Modified RRT (MRRT), RRT*, and Batch Informed Tree (BIT*).

Optimization-based planning involves formulating the path-planning problem as an optimization problem with single or multiple objective functions. The goal is to find the optimal path that satisfies a set of constraints. The most common optimization-based planning methods include gradient-based optimization [6], iterative optimization [7], and mixed-integer linear programming [8].

While some research groups have focused on particular applications such as biopsy, ablation, drainage, deep brain stimulation, and Stereo-electroencephalography, others considered a broad scope of applications and proposed a surgical tool-specific algorithm. The different needle-like surgical tools used for minimally invasive neurosurgical procedures include bevel-tip flexible needles, steerable and trocar needles, catheters, guidewires, stents, probes, and suction devices. 

Through this systematic search review paper, we aim to review the literature on relevant algorithms for automatic or semi-automatic path planning for neurosurgical interventions based on the intervention tool type, application type, and the scope of the validation study (i.e., simulation, phantom, preclinical, and clinical studies). We will also summarize and report the most recent advancements in the field and future trends in path planning for neurosurgical applications. In the following sections, we will outline the organization of the paper.

### 1.2. Related Work

Starup-Hansen et al. [9] conducted a systematic review of automated path-planning methods for stereotactic brain tumor biopsies focusing on artificial intelligence (AI)-based algorithms. Moreover, a most recent review paper focusing solely on straight tool path planning, published by Zanello et al. [10], covers literature up until September 2019 and includes 42 papers. Ye et al. [11] published a review paper in 2024 that reports different path-planning methods for bevel-tipped steerable needles. However, this is a technical paper and does not focus on neurosurgical applications, limitations, and challenges. To the best of our knowledge, and based on our systematic review, this is the first review paper published in the last decade that reports on various neurosurgical path-planning techniques. This paper not only includes straight tool path planning but also addresses automatic path planning using steerable needles and concentric tube robots, which are currently under development for minimally invasive neurosurgical applications.

### 1.3. Our Contributions 

Our review paper presents three novel aspects compared to recently published review papers as follows: (1) it examines publications from the past 12 years; (2) it addresses three types of interventional tools: straight tools, steerable needles, and concentric tube robots; (3) it focuses on path-planning algorithms specifically developed and tested for neurosurgical applications. Table 1 provides a summary of this comparison.

### 1.4. Organization of the Paper

**Figure 1:**
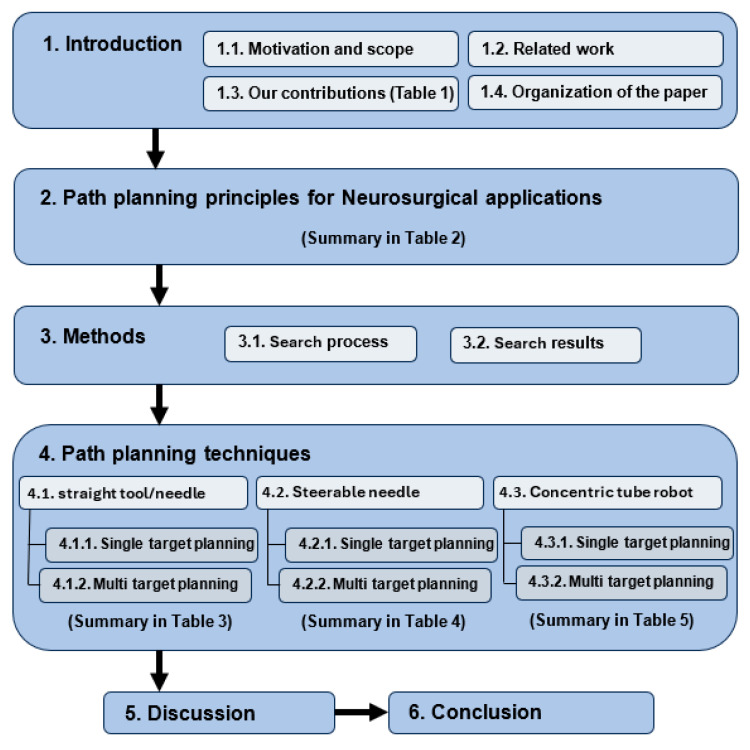
Diagram outlining the organization of the paper.

We have covered the path-planning principles in Section 2, our review method in Section 3, findings in Section 4, and concluded this paper in Section 5. In Section 4, we discuss path-planning techniques for three types of intervention tools: straight tools (Section 4.1), steerable needles (Section 4.2), and concentric tube robots (Section 4.3). Each study was individually analyzed for anatomical region of interest, path-planning technique employed, criteria for using the specific path-planning technique, type of imaging used, type of tool used in the study (needle, endoscope, etc.), type of trial (phantom, animal, human, etc.), preoperative planning vs intraoperative planning, manual or automatic segmentation, and study assumptions. In addition, we compared the different path-planning algorithms based on the limitations and the primary outcome measures (if provided). We also summarized each section with key points in a table. Figure 1 shows the organization of this paper.

## 2. Path-Planning Principles for Neurosurgical Applications

Minimally invasive neurosurgical procedures, also called image-guided keyhole or stereotactic neurosurgery procedures, are based on pre-operative or intraoperative CT/MRI images and performed through a small burr hole on the surface of the skull.

**Figure 2:**
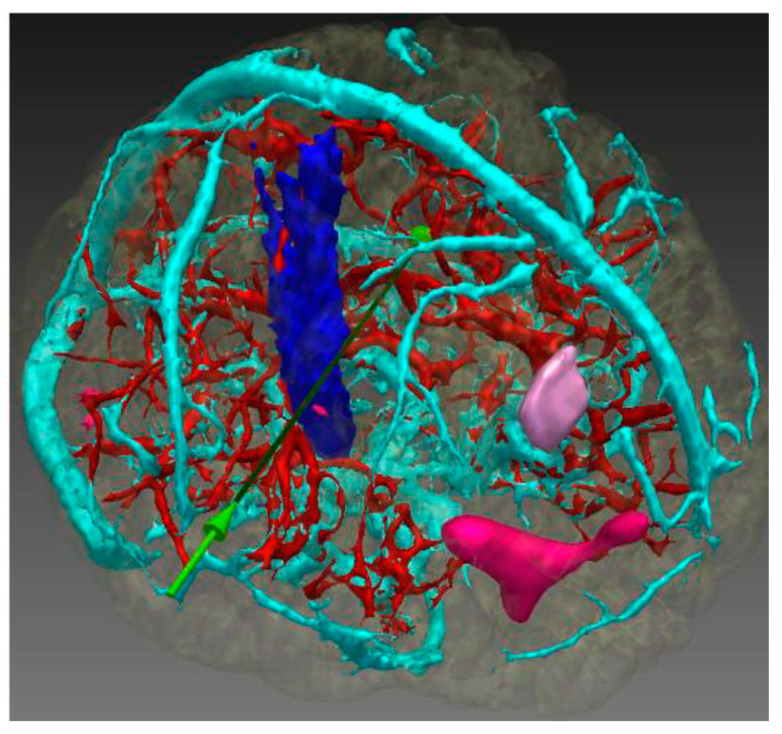
Path-planning example showing trajectory and critical structures. Blue: cerebrospinal tract (CST); pink: hypometabolism PET; deep pink: SPECT; cyan-red: blood vessels; green arrow: historical trajectory [16].

Path planning is a primary workflow step for all image-guided keyhole neurosurgeries. Path planning for percutaneous interventions involves finding a safe and feasible/optimal path for needle insertion by avoiding obstacles within the body to reach the targets defined by a clinician or a computer-based algorithm. Obstacles or critical structures refer to any tissue that cannot be damaged or punctured by the needle such as bone, nerves, or arteries. Figure 2 shows different critical structures segmented on an MR image dataset. A target can refer to a planned implant location of a seed, a point within a tumor for biopsy and ablation, or the appropriate distal location of an electrode in the brain for DBS.

During DBS multiple stimulation electrodes are stereotactically implanted in specific brain targets, including deep gray matter nuclei. DBS is considered a treatment option when neurological disorder symptoms, such as motor symptoms in Parkinson’s disease, are resistant to drug therapy. In this treatment, an electrode is placed deep in each hemisphere of the brain to target a certain nuclei (a few millimeters long targets) through a hole drilled in the skull. The electrode is then energized using a pacemaker to stimulate the nucleus with high-frequency impulses. Improving the clinical outcome of the treatment requires optimization of the trajectory as well as the target point. For instance, Dergachyova et al. [12] developed a path planner that optimizes both the trajectory as well as the target point. It should be noted that in this paper, our primary emphasis will be on the trajectory-planning aspect, and target planning will not be discussed since we assume that the target point or region of interest is identified by the neurosurgeon.

Epilepsy is a brain disorder that causes recurring seizures. The epilepsy incidence rate is 5–6/1000, while 30% of them are refractory to medication [13] and require minimally invasive intervention. Minimally invasive treatment of epilepsy requires two steps. The first step is a diagnostic procedure called Stereo-electroencephalography (SEEG), a replacement technique for subdural grid placement (SDG), and the second step is an ablation treatment procedure. SEEG is a minimally invasive surgical technique that involves the implantation of multiple-depth electrodes into the brain. These electrodes are placed stereotactically, meaning they are guided by a three-dimensional coordinate system based on 3D imaging studies such as MRI or CT, through small burr holes in the patient’s skull. The primary goal of SEEG is to record electrical activity from deep brain structures to identify the precise origin of epileptic seizures. SDG involves the placement of electrode grids or strips directly on the surface of the brain (cortex) through a craniotomy, which is a surgical opening of the skull. These electrodes are placed subdurally, meaning they lie between the brain’s surface and the dura mater (the outermost membrane covering the brain). As opposed to the SEEG, the primary goal of SDG is to record cortical electrical activity and identify the regions responsible for seizure onset.

Once the regions are identified, a separate minimally invasive laser ablation procedure may be scheduled to ablate the region to eliminate the seizure source. Path planning for these procedures, specifically the diagnostic phase, is very time-consuming. Path planning for multiple targets requires a multi-target patsh planner. Other neurosurgery applications also could be either single-target or multi-target procedures. Therefore, in this paper, both single-target planners and multi-target planners will be investigated. 

**Figure 3:**
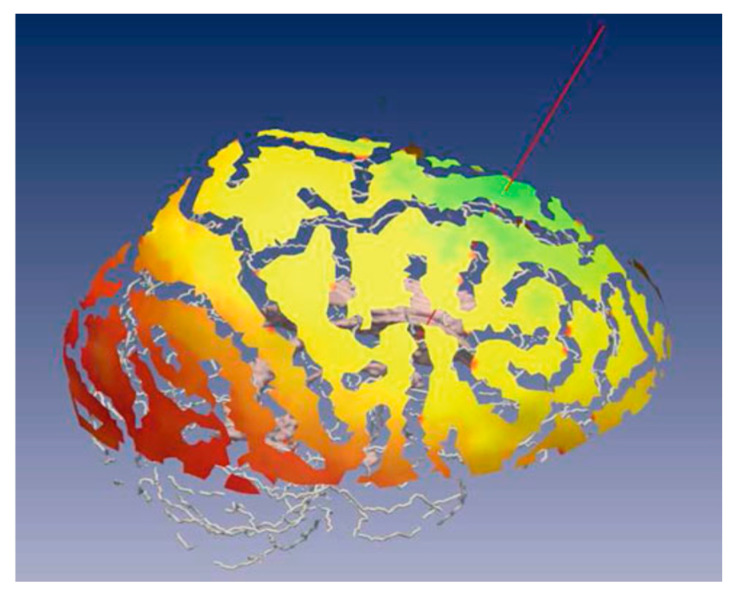
Color map of soft constraint on the skull of a patient generated for guiding the neurosurgeon to select the best entry point. Green region are safe entry points, yellow region are entry points with moderate risk, are red region are high risk entry points and are No-Go points. Reprinted/adapted with permission from Ref. [15].

For path planners in minimally invasive procedures, the path planner should search for an appropriate entry point and an optimal trajectory. When the target is a single point and the tool is a straight, the path planning reduces to an entry point planner. Therefore, in the case of the straight needle, once the entry and target points are planned, the path is fully defined. For the straight needle scenario which covers the majority of the clinical procedures, the path planner could be developed to either output the best entry point on the skull or function as a decision-support system by visualizing color-coded regions on the skull labeled as “safe regions”, “low-risk regions”, and “high-risk regions”. This accessibility map could help guide neurosurgeons to manually select the entry point [14,15,16,17,18,19]. Figure 3 shows an example of accessibility map, with the green region labelling the safest region to plan the entry point. When the tool is curved, on the other hand, the path planner should plan the entry point as well as the curvilinear surgical tool trajectory to fully define a safe and optimal path to reach the desired target.

To consider implementing a software package as clinical decision support software (CDSS), the software should meet the following criteria: (1) visualize medical images and analyze, or print medical information, (2) provide recommendations to neurosurgeons regarding prevention, diagnosis, or treatment plans, (3) not be intended to acquire, process, or analyze medical images, and (4) the neurosurgeons must be able to have access to review the basis for optimal trajectories automatically generated and recommended by the software. Safe and optimal trajectories avoid certain critical structures and optimize certain parameters as listed in Table 2.

**Table 2:** Structures that should be avoided and parameters to optimize.

	Penetration to the midline
	Blood vessels
	Critical areas of the cortex, such as those responsible for upper and lower limb motor system, speech, and sensory function [16,18]
	Small vessels at the fundus of the sulcus [13] (this is especially critical in the absence of angiography) [12],
	Arachnoid stretching
Avoid	Any neighboring electrodes (in the case for SEEG procedures) [20],
	Ventricles
	Nerve tracts [21].
	Low quality SEEG recordings [22],
	Minimize overlap with caudate [17]
Optimize	Maximize overlap with gray matter [17,20].

Other considerations include the fact that the entry point should be posterior to the hairline for cosmetic reasons and preferably not through the motor cortex to avoid side effects [23]. The intracerebral length of the path should be minimized [15,20]. The drilling angle to the skull and sometimes the orientation of the electrode should be optimized depending on the target shape [15,20]. 

Trajectory-planning algorithms have been developed for deep brain stimulation (DBS) electrodes [15,17,24,25], biopsy needles [19,26,27,28,29], and SEEG electrodes [13,22,30,31]. These methods provide either (1) assisted planning to aid manual trajectory selection [27,28,31]; (2) automated planning for a single trajectory planning [15,16,17,25,29,30,32,33]; or (3) automated multiple trajectory planning [13,22,30,31,32]. 

Different research groups have developed automated single trajectory-planning algorithms to maximize distance from blood vessels while satisfying other surgical constraints for individual trajectories [15,17,19,29,34]. Each method defines a unique risk score based on the proximity of the trajectories to vessels and other critical structures. Trajectories closer to critical structures are assigned higher costs, and ultimately, the trajectory with the lowest costs is selected and recommended to the neurosurgeon. In most of these approaches, surgeons manually specify a target point or target region [16,17,19,29,33,34,35]. 

For instance, in the approach by Sparks et al. [32], the neurosurgeon must define a set of targets as single points and suggest a possible entry region on the skull. The path planner then generates the optimal path based on maintaining a safe distance from critical structures, maximizing gray matter sampling, and ensuring a safe distance between electrodes for multi-electrode placement. Zelmann et al. [31] relaxed the need to define specific target points by selecting the entire hippocampus and amygdala as regions of interest (ROI). Targets were then selected through Gaussian distribution sampling, resulting in sampling the targets close to the ROI centerline. The optimal trajectory is calculated based on a reasonable entry angle on the skull, a safe distance from the critical structures, and penetration optimization for elongated targets such as in the hippocampus [33]. Sparks et al. [33] targeted a hippocampus for epilepsy treatment, planning multiple point targets for SEEG electrode placement to identify seizure points, rather than placing a single electrode along the entire hippocampus.

Avoiding obstacles is the most computationally demanding part of path-planning algorithms [36]. To estimate safe trajectories, Bériault et al. first assessed potential paths by eliminating those within an unsafe distance from critical structures. They then evaluated the remaining trajectories using a weighted sum of (1) distance from critical structures and (2) the total distance of points along the needle relative to all critical structures [24]. Vaillant et al. [34] summed traversal costs to estimate a risk score, assigning higher costs to critical regions. Similarly, another approach [35] used a cost function based on the summed traversal costs along the trajectory and the minimal distance of the trajectory from blood vessels to reduce the risk of hemorrhage.

Shamir et al. [19] computed each pixel’s risk score considering the distance to critical structures to determine (1) the shortest distance to the critical structures associated with the maximum risk and (2) the total sum of the risk along the trajectory. Essert et al. [15] proposed a generic optimization-based algorithm for path planning. This method involves defining a set of hard constraints for high-risk critical structures, and soft constraints that allow for optimization. The trajectories that violate the hard constraints are eliminated, and the optimization algorithms are applied to the remaining poll of trajectories. To account for regions for motor, somatosensory, speech, and vision functions during automatic path planning, Trope et al. [29] proposed a patient-specific physiological mapping of the brain regions. This secondary measure allows for the evaluation of the initially generated safe and optimal path based on the possible risk to neural function. They assigned high weights for distance cost to these structures and lower cost to white matter (WM) tracts and cortical function regions, creating trajectories through these areas only if no lower-cost path existed. For instance, Liu et al. [23] use a cost function that penalizes trajectories passing less than 1 mm through the thalamus. Marszalik et al. [37,38] developed a path assessment algorithm that quantifies the damage to different structures in the brain along the needle trajectory.

## 3. Methods

### 3.1. Search Process

We searched Google Scholar, PubMed, IEEE explore, and Scopus for articles about path-planning techniques published between January 2012 and July 2024. Google Scholar provides a unique and comprehensive resource for scholarly research. According to one study, the size of Google Scholar was estimated at 100 million documents as of September 2014, covering about 88% of all scholarly documents accessible on the web in English [39] and this number is exponentially growing. Another study estimates the Google Scholar database to be 389 million as of 2019 [40]. 

We used the following keywords to search the scholarly databases:

Dataset A = {Trajectory planning brain, Path planning Brain, Planning brain, Planning neurosurgery, Planning neurosurgical, Automatic planning Brain}

Then, we excluded duplicate studies, those unrelated to path planning, and those unable to be retrieved. We then excluded papers with the following keywords in the title:

Dataset B = {Treatment planning, Virtual reality, 3D printing, Augmented Reality, Mobile robots, Automatic segmentation, Care planning, Planning target volume, Radiotherapy planning, Radiosurgery}

We also excluded reviews, editorials, lectures, case studies, protocols, studies written in languages other than English, abstracts, and retracted papers. The review team consisted of three medical students, robotic experts with expertise in path-planning techniques and medical robotics, and a board-certified neurosurgeon. The review team investigated the search results based on specific inclusion and exclusion criteria. Studies were excluded based on non-percutaneous interventions, use not particular to any anatomical region, and no path-planning methods described. We used the Preferred Reporting Items for Systematic Reviews and Meta-Analyses extension for Scoping Review (PRISMA-ScR) statements to strengthen the methodology [41]. We used Zotero [42] and Harzing’s Publish or Perish software [43] to export, organize, and analyze the papers identified by the initial search.

### 3.2. Search Results

**Figure 4:**
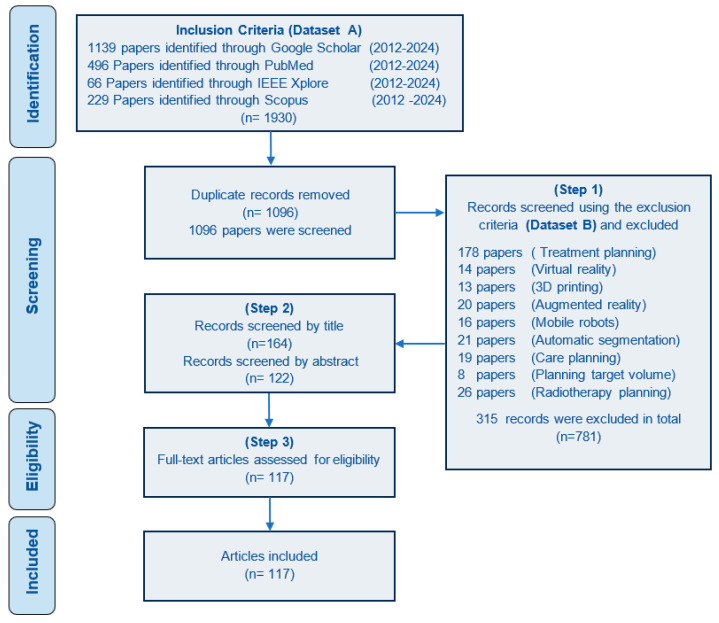
This diagram shows the screening process at every stage of the literature review.

Figure 4 shows the data extraction results at every screening step. 1096 study titles were obtained from initial database search using keywords listed in Dataset A after removing the duplicated papers. We excluded 315 that met at least one of our exclusion criteria listed in Database B, leaving 781 studies to be assessed by screening the title and the abstracts. This screening step resulted in 122 papers and after the eligibility check, we included 117 papers in this review paper.

**Figure 5:**
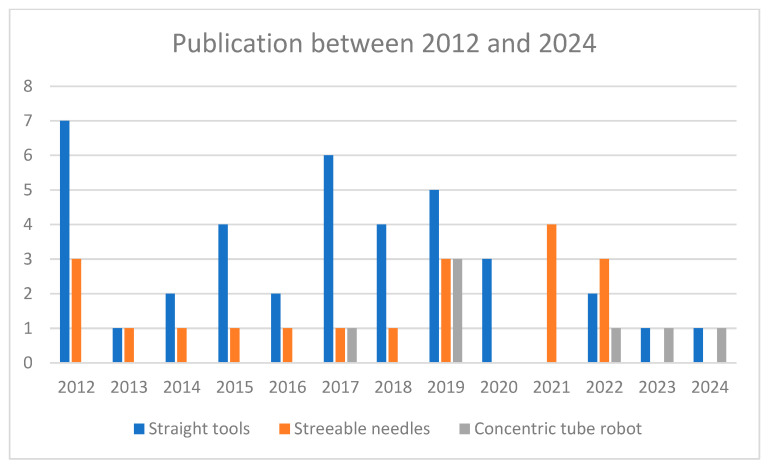
Annual number of publications in three different categories.

Figure 5 illustrates the number of publications that investigated automatic path-planning algorithms for three categories: (1) straight tools, (2) steerable needles, and (3) concentric tube robots, from January 2012 to July 2024. In the following sections, we have discussed these papers in detail. 

## 4. Path-Planning Techniques

### 4.1. Path Planning for Straight Tool/Needle

#### 4.1.1. Single Trajectory Planning

A. Graph search techniques

There are several graph search techniques, namely Brute Force search, Breadth First, Dijkstra, and A*. For more information about these algorithms see [43]. Brute Force search exhausts all the points on the selected part of the skull as possible entry point candidates to safely reach the given target point or target region. Therefore, this technique grantees the optimal solution based on the specific cost function and within the given skull region [17]. Several research groups including our team have used this brute force search algorithm for path planning [44,45,46]. A team at University College London developed a visualization software called EpiNav [16] and implemented different path-planning algorithms for straight and curved tools. For straight tool planner, they developed a brute force-based entry point search algorithm and implemented it on the graphics processing unit (GPU). 

The algorithm takes the selected part of the skull mesh as the input and processes all the vertices to search for the best entry point, considering the maximum of ten degrees as entry angle (angle between the skull normal vector and the trajectory connecting the entry point and the target point) [16]. Trajectories perpendicular to the skull are preferred since, during the drilling phase, the surgical tool is less prone to possible slipping and deviating from the planned trajectories [22].

For automatically planned trajectories, the needle length, drilling angle, minimum distance from critical structures, accumulated risk (cumulative distance from critical structures), and minimum trajectory distance from brainstem were calculated. Due to lack of vascular imaging, the blood vessels were not considered as a critical structure. Instead, sulcal models were used as proxies for these critical structures, assuming the blood vessels would most likely to be present within sulci [47]. The search time depends on the number of vertices in the skull model. 

Favaro et al. [48] used a brute force search method within the circular possible entry points area on the skull, defined by a neurosurgeon. They implemented their path planner using open-source 3D Slicer software for visualization. They tested their path planner using a sheep (animal) model. 

Liu et al. [25] conducted a multi-surgeon, multisite brute force-based path planner validation study for deep brain stimulation procedures, both retrospectively and pseudo-prospectively. Out of 60 trajectories, their automatically generated trajectories were accepted by most neurosurgeons in 95% of the cases. Cost function weights were heuristically and iteratively estimated based on surgeons’ input. First, the surgeon set the initial weight values. Then, automatically generated and manually selected trajectories were presented to the surgeons while they were blind to how the trajectories were generated. If the trajectory was considered preferred, it was classified as “Excellent”. If the surgeon could not confirm if a presented trajectory was superior to their manually planned trajectory, it was rated as “Equivalent”. If the presented trajectory was not preferred as superior but could still be considered clinically viable trajectory, it was labeled “Safe”. Finally, the surgeon rejected, it was considered unacceptable. Then, the weights were manually adjusted based on the feedback, and the experiment was repeated until each surgeon ranked all the training trajectories as at least acceptable. Once these parameters were estimated, they were fixed and used for all evaluation experiments.

Vakharia et al. [49] also conducted a multicenter study for selective laser amygdalohippocampectomy. Based on this study, blinded external experts were significantly more likely to prefer automatically generated trajectories to manually planned trajectories. This planner maximizes ablation of the mesial hippocampal head and amygdala, while sparing the para hippocampal gyrus using a machine learning-based approach utilizing random forest and linear regression [50]. 

Hamz’e et al. [51] used their previously developed path planner [15] to factor in the intraoperative brain shift to automatically adjust the plan. This patient-specific automatic path-planning method uses an optimization algorithm and Finite element method (FEM)-based brain shift simulation to simulate the brain shift and generate an optimal path. They compared the results with paths generated without considering the brain shift. The results illustrated the safe insertion zones (recommended safe zone to neurosurgeons for manual selection of the entry point) for DBS interventions shrunk by more than 50% when considering a possible brain shift. Segato et al. [52] also developed a position-based dynamic simulator to estimate brain deformation allowing for more accurate path planning. The validation study results, using recorded deformation data of in vivo animal trials, illustrated a close match with real brain deformations with a mean mismatch of 4.73 ± 2.15%.

B. Sampling-based techniques

Sampling-based solutions are the current trend for generic single-query, path-planning problems [53]. This technique is centered around random sampling of the skull points as possible entry point candidates.

Different retrospective studies have shown the efficacy of automatic path planning for straight needles. Trope et al. [29] conducted a retrospective study on eight patients. They investigated three different path-planning techniques: (1) manual path planning, (2) augmented visualization for manual path planning, and (3) automatic path planning. This group considered ventricles, blood vessels, white matter fibers tractography, and functional (motor, sensory, speech, and visual) areas as critical structures. Based on their proposed technique, 1000 candidate entry points on the related skull region were deterministically sampled, given the predefined target. Then, an accumulated risk value was computed along each candidate trajectory (e.g., a line between the entry and target points pair). The path planner outputs three trajectories of minimal risk values at least 2 mm apart. The surgeon then selects the best trajectory through a visual inspection of these three proposed trajectories. A total of 120 surgical trajectories were collected (five surgeons, eight targets, three methods) and analyzed. The automatically planned trajectories resulted in improved risk scores by 76%; the average distance from nearby blood vessels to the planned trajectory increased by 1.6 mm (SD = 0.5, *p* < 0.05) from 0.6 to 2.2 mm (243%). A similar technique was used by Zelmann et al. [13,31]. In addition to considering critical structure constraints, they optimized based on maximizing the (1) recording of target volume, and (2) recording of gray matter. Based on a surgeon’s feedback, 95% of the automatically planned optimized electrode trajectories would likely be considered for clinical use. Regardless of the path-planning method, different techniques could factor in indecision-making criteria and constraints to develop a path planner. The planners work based on considering two types of constraints: (1) soft constraints, and (2) hard constraints [12]. Hard constraints in neurosurgery path planning are strict rules, like avoiding vital structures. Soft constraints are flexible guidelines, such as minimizing tissue damage. Balancing both ensures safe and effective surgical paths. Any trajectory that violates the hard constraints, such as those intersecting with vessels or the brain’s mid-plane, is removed from the poll of initial trajectories. However, the path planner should optimize the soft constraints by minimizing a cost function. The soft constraints (including distance from different critical structures, maximum penetration to gray matter and target region, minimum penetration to white matter, and minimum needle length) should be collectively optimized to achieve the best trajectory. Some research groups also investigate path planning using only soft constraint concepts with proper weight tuning. Hackenberg et al. [54] showed that this approach has two advantages: First, it significantly reduces the computation times. Secondly, it gives more control to the surgeon to select from a larger pool of admissible paths. This results in a multi-objective optimization problem and requires searching for the best compromise between these constraints. This problem is usually addressed by considering a nonobjective cost function defined as an aggregative weighted sum of all the constraints.

**Figure 6:**
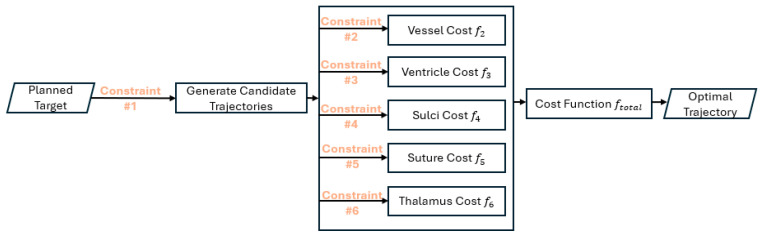
Path-planning flowchart for multi-objective cost function. Reprinted/adapted with permission from Ref. [23].

Figure 6 shows a flowchart for a path-planning algorithm using a multi-objective cost function. The weights are assigned based on consensus of the experts in the field or automatically through a learning process from Post-operative Images [14], or weight-planning approach to reach increase similarity of automatic path-planning results to manually planned trajectories by the neurosurgeon [55]. If one of the terms is required to be maximized in the cost function, the weight of that specific terms must be negated [48]. However, Hamze et al. [56,57,58] developed a multi-objective dominance approach based on Pareto Front technique. They compared the multi-objective dominance approach to a classical aggregative weighted sum of the multiple constraints through a retrospective study performed on 14 DBS cases. The results show that the former method covers a larger choice of relevant optimal entry points (optimal trajectory, defined by the assigned entry point and given target point) than the classical weighted sum approach. They showed that the classical weighted sum discards some of the possible trajectories that the neurosurgeon might preferred. Another group used a fuzzy logic technique to assess the risk factor for candidate trajectories. The advantage of their proposed algorithm is that it reduces the recommended safe insertion area based on the operation [18,59].

Tuning the weights for objective cost function is nontrivial because neurosurgeons have no consensus about the optimal trajectory [23]. Liu et al. [23] developed a surgeon-specific automatic path planner that outputs trajectories that a certain surgeon prefers after training. For each neurosurgeon, the cost function is uniquely tuned. They initially set the weighting factors based on the specific surgeon’s suggestion and then manually adjusted the weights until the surgeon is satisfied with all 20 automatically computed trajectories. To test the path planner, they conducted a blind validation study by instructing the neurosurgeons to choose a clinically preferable path between their manually planned path and automatically generated path while they were blinded to the identity of the paths. This study showed that in 10 out of 40 cases, neurosurgeons preferred the automatically computed trajectory over their own. In 27 of the remaining cases, the computed trajectory was rated to be equivalent to their own manually planned path or otherwise acceptable.

C. Optimization-based methods

Dergachyova et al. [12] used a multi-objective cost function and optimization-based path planner to plan a trajectory for DBS and optimize the target point location and the trajectory to reach from the entry point to the target point.

Villanueva-Naquid et al. [60,61] proposed a preoperative assessment to find the safest trajectory in keyhole neurosurgery using genetic algorithm. This method optimizes the sum of maximum risk values to achieve the safest trajectory. This algorithm is designed based on Darwin’s fittest principle of survival [62]. This technique decreases the computation time by 99.9% compared with an exhaustive search. The path-planning results were validated by a group of neurosurgeons. For more information on artificial intelligence-based optimization approaches refer to [9]. 

#### 4.1.2. Multiple Trajectory Planning

For epilepsy procedures, often 10 to 15 electrodes are implanted in the brain, which means that multiple path plannings are required. Each electrode requires a unique entry point and a target point. Theoretically, all the techniques introduced for single trajectory planning in early sections could be adopted for multiple trajectory planning with minor considerations. 

Multiple trajectory planner should consider the quantitative measures for each individual trajectory and the fact that electrodes must not contact each other. Therefore, each trajectory should be considered a critical structure for other trajectories to avoid interference.

Sparks et al. [33] developed a multiple trajectory path planner for epilepsy procedures based on EpiNav. Once the neurosurgeon selects the target points within each ROI, electrode trajectories are automatically computed in two steps: (1) trajectory risk scoring based on a cumulative distance between the trajectory (defined by an entry point on the skull to the target point within the brain) and critical structures, (2) implantation plan computation to determine a feasible combination of low-risk trajectories for all electrodes. Their algorithm uses dynamic programming to reduce the search space and a depth-first search to find all surgically feasible trajectories that do not intersect with other trajectories or critical structures while maximizing the gray matter sampling. 

This method was evaluated retrospectively on 20 patients (190 electrodes). The study showed their method lowered the quantitative risk score in 83% of electrodes. This method found suitable trajectories for 70% of electrodes that offer similar success rates compared to manually selected trajectories. When sulci were removed from the list of critical structures, the success rate for the automated path-planning technique was increased to 95%. The average computation time for this technique was 54.5 (17.3–191.9) s when computing between 7 and 12 trajectories. In this study, the neurosurgeon specifies the ROI. Target points are then automatically sampled in this region to capture grey matter (GM) within and near the medial surface or to capture deep GM within the ROI. Target points are clustered if multiple electrodes are placed in the same ROI to ensure unique targets for each electrode. Some hard constraints (Maximum length and entry angle) are relaxed if the path planner cannot generate suitable trajectories within the given constraints. 

Another retrospective study by the same team [32] was conducted assuming that the neurosurgeon directly specifies the target points. They compared their multi-target planning to (a) manual path planning and (b) a single trajectory-planning algorithm neglecting collision between electrodes on 18 patients with 165 electrodes. Their multi-target planning algorithm is computationally efficient as it requires 1 min to plan trajectories for 7–12 electrodes compared to 2–3 h for manual planning. The multi-target planner changed all 165 trajectories compared to the manual plan. These changes resulted in lower risk (122) and increased grey matter sampling (99). It provided a shorter penetration length for 92 electrodes and improved the entry angles for 113 electrodes. The multi-target planner changed 42% (69/165) trajectories compared to the single path planner to resolve electrode conflicts. These promising results set the ground for the first prospective study by Vakharia et al. [20] from the same institute in 2019. 13 patients (125 electrodes) were prospectively recruited to undergo SEEG. This study was the first prospective study of its kind. EpiNav (UCL, London, UK) [16] was employed to run the path planner to automatically generate optimal trajectories. The generated trajectories in 30% of the time resulted in significantly lower risk scores than those manually generated. They also externally validated their path planner by hiring five independent, blinded experts from outside institutions [63]. They extended the application of their path planner to stereotactic brain biopsy and retrospectively compared the result of the automatic path planner and manual path planner on 15 patients. Mean angle determined using the automatic path planner was more perpendicular to the skull than using the path generated manually (10.0° vs. 14.6° from orthogonal), the mean trajectory length was shorter (38.5 vs. 43.5 mm), and the risk score was lower (0.27 vs. 0.52; *p* = 0.03) [64].

Vakharia et al. [65] also used EpiNav to successfully generate multi-trajectory plans capable of laser interstitial thermal therapy (LiTT) anterior two-thirds corpus callosotomy. Their automatic path planner significantly improved the safety metrics compared to external expert manual plans based on a blinded validation study. Residual Interhemispheric connectivity was 1 out of 10 cases for computer-assisted planning, 4 out of 10 for manual path planning by expert 1, and 2 out of 10 cases for manual path planning by expert 2 estimated based on probabilistic tractography.

**Figure 7:**
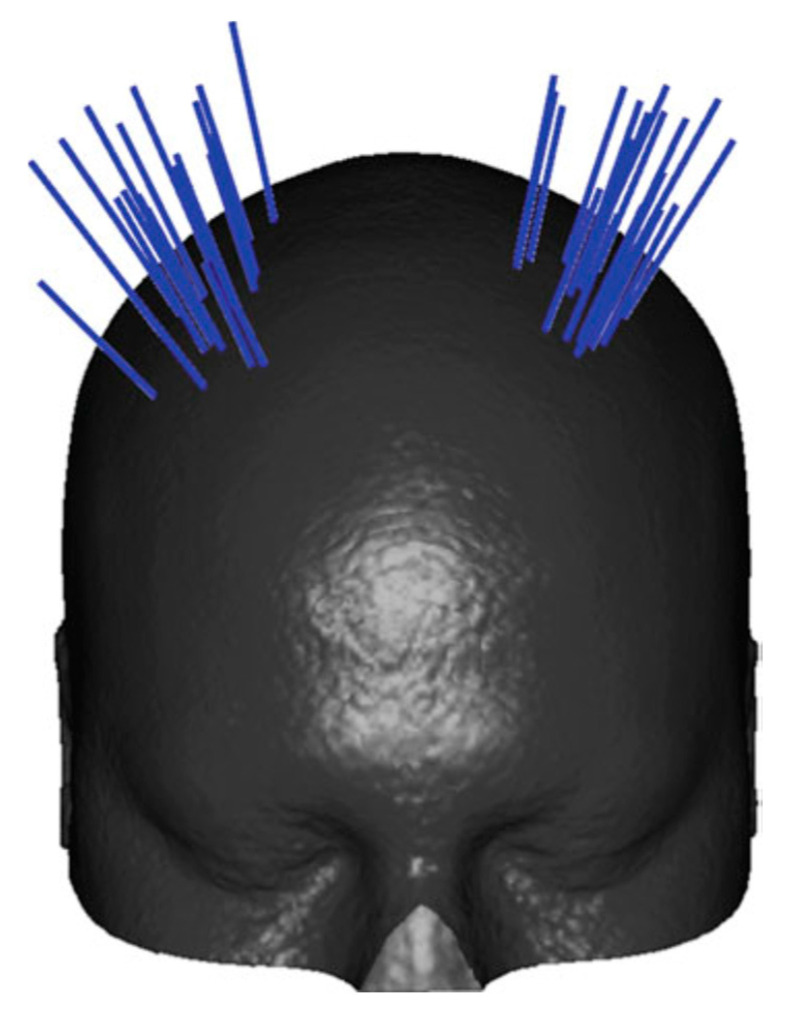
Atlas of deep brain stimulation procedure of the left and right subthalamic nuclei for patients treated between 2009 to 2011. Reprinted/adapted with permission from Ref. [17].

Momi et al. [30] also developed a multi-trajectory planner based on a brute force search (an exhaustive search). They evaluated the path planner both quantitatively and qualitatively. For the qualitative study, they performed Fleiss’ kappa analysis to assess the four surgeons’ agreement on the 26 trajectories based on “good”, “acceptable”, or “discarded” for each considered method (*p* < 0.05). This team later improved their method by using an atlas to limit the search space to the anatomical structures in which the neurosurgeon placed the entry points and the target points respecting the epileptological strategy for the implantation. Secondly, they proposed a new selective brute force approach to improve computation time implemented in 3DSlicer [66]. An example of an atlas for deep brain stimulation procedure is shown in Figure 7 [17].

Computation time for multi-target planning could be longer than single-target planning. However, since the computation for each path could be performed independently [29], with some fine-tuning to avoid collision between electrodes, parallel processors such as GPUs could be very useful in improving the planning time. A study by Rincón-Nigro et al. [29] showed that GPU-accelerated path computation could be two orders of magnitudes faster than CPU-based computation. Choosing the computation and visualization software is critical for implementing a path planner for GPU-accelerated computing. For instance, 3D slicer does not provide the possibility of multi-core parallelization or GPU acceleration [66]. Table 3 summarizes publications related to straight needle trajectory planning. 

### 4.2. Path Planning for Steerable Needles

There are different types of steerable needles. Beveled-tip needles naturally bend while pushed into tissue due to the tip asymmetry and lateral force. These needles with a fixed-shape bevel tip could be steered by axial rotation of the whole body could result in large stresses on the tissue due to the curvilinear path [67].

**Figure 8:**
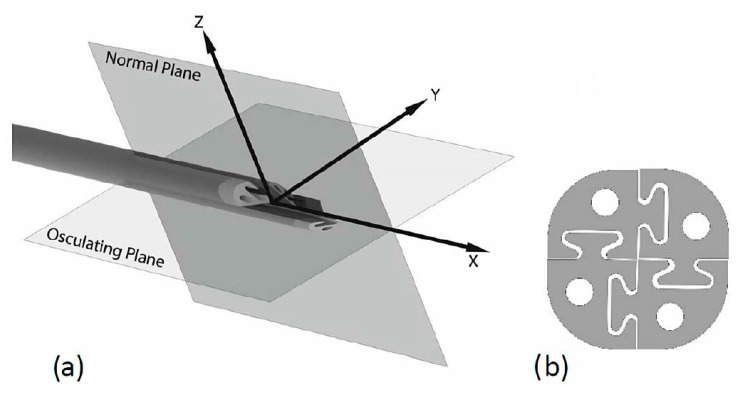
STING is made of 4 identical quarters (**a**), STING cross-section with interlocking mechanism is shown (**b**). Reprinted/adapted with permission from Ref. [71].

In Europe, there has been a multi-institutional endeavor, Enhanced Delivery Ecosystem for Neurosurgery 2020 project (EDEN2020), funded by the European Union’s Horizon 2020 research and innovation program (www.eden2020.eu, accessed on 9 May 2024), to develop a gold standard method for one-stop diagnosis and minimally invasive treatment in neurosurgery. They have developed a novel steerable needle that consists of four identical quadrants as shown in Figure 8. This biologically inspired steerable needle system, initially called STING, was proposed to tackle challenges associated with conventional needle steering systems, including tissue trauma, and to improve the workspace and applicability [67,68]. Pinzi et al. [69] proposed a GPU-accelerated computer-assisted planning algorithm for steerable needle insertions.

This path planner generates optimized curved 3D trajectories, maximizes ablation of the hippocampi complex, and minimizes the collateral damage to nearby structures. They tested their path planner on five patients with mesial temporal sclerosis. This is the first clinical application of preoperative planning for steerable needle-based LiTT. This study showed the promise of improving LiTT procedure efficacy by improving safety and increasing the ablation zone.

Path planning for all these steerable needles follows the same principle with special consideration for the unique kinematic constraints of each tool. This section covers different path-planning techniques for steerable needles regardless of their specific kinematic constraints. 

#### 4.2.1. Single Trajectory Planning

Brute force search, Breadth-first search, Dijkstra’s algorithm, and A* are four typical graph-search methods based on the discrete approximation of the planning problem. These techniques are relatively simple but computationally expensive due to the high dimensional search space for steerable tools. Therefore, they are not widely used in neurosurgical applications for path planning of steerable tools. Pehlivanoğlu et al. recently developed a path-planning framework for the first time that handles tracts and atlas-based segmentation for path planning [70]. Their framework includes Dijkstra’s and A* path-planning algorithms. However, the proposed framework is dynamic and flexible and can handle different path-planning algorithms.

As discussed previously for curved tools, the path planner should output the best entry point as well as the optimal trajectory associated with the planned entry point. Path-planning methods for curved tools include different types of sampling-based path planners such as RRT, RRT*, and BIT*.

A. Sampling-based techniques

*Rapidly exploring Random Trees technique*—Chiara Caborni et al. [72], a research team from Imperial College, developed a path-planning approach for neurosurgical applications based on well-known Rapidly exploring Random Trees (RRT). This path-planning approach can produce a curvature-constrained path, which is required for steerable needles to address the kinematic constraints challenge, in complex brain risk maps. This multi-tree approach ensures that several solutions are generated so that the clinician can evaluate and select a path that minimizes the cost of different weighted competing objectives, such as the length of the path, the clearance from vessels or nervous bundles, and most importantly, the accumulated risk in crossing different brain regions. This technique results in a “path of least risk” for the patient. This approach was tested in simulation and the reported maximum error is constrained to 2 mm. 

**Table 3:** Summary of publications on straight needle path planning.

Authors/Year	Application	Path-Planning Method	Objectives	Evaluation Method	Critical Structures	Imaging Method	Tool Type/ Accuracy/ Efficiency	Assumptions/Evaluation Measures/Other Descriptions	Group/University Name
Essert et al. [14,15], 2012; ([55], 2015) ([36], 2018)	DBS	Extensive search over the parameters space, (Bidirectional RRT)	Automatic cost function weightage optimization	Retrospective study (N = 1) (N = 56) (N = 16)	Cortical sulci, and ventricles	T1-weighted MRI (MRI) (CT)	Straight tool	Use knowledge from surgeon’s previous path planning to tune the weights. This method could be used with any linear multi-objective cost function	Université de Strasbourg
Liu et al. [23], 2012 (2014 [25]) (2019 [4])	DBS (neurosurgery)	Exhaustive search within boundary selected by surgeon	entry point optimization	retrospectively and then pseudo prospectively, Iner surgeon comparison (T = 40)	Blood vessels, ventricles, thalamus, cortical surface,	MR T1 with and without contrast (Combined MRI and Diffusion Tensor Imaging, DTI)	Straight tool 92.5% success rate by two surgeons	Weighting values were effectively tuned to mimic the planning preferences of individual surgeon Used Waypoint Navigator planning system	Vanderbilt University
Bériault et al. [17,73], 2012	DBS	Exhaustive search of search space using Voxel-based cost map	Path planning without using contrast enhanced MR images	Retrospective (N = 8)	Entry point within the frontal lobe and avoid the midline (hard constraint). Avoid ventricles and sulci (hard/soft constraint) Avoid subcortical blood vessels and minimize overlap with caudate and cortical gray matter (soft constraint).	multi-modal MRI analysis (T1w, SWI, TOF-MRA)	Straight tool	Venous and arterial vessels segmented using dense susceptibility-weighted imaging (SWI) and TOF-MRI An entry point should be selected within the frontal lobe and anterior to the primary motor cortex.	McConnell Brain Imaging Centre, Montreal Neurological Institute, McGill University,
Herghelegiu et al. [27], 2012	Brain Tumor Biopsy	Identify stability map (safe regions on the skull as entry points)	Provide an assistive tool	User study	Blood vessels	MRI	One neurosurgeon and two neurosurgery residents tested and provided positive feedback		Vienna University of Technology
Shamir et al. [19], 2012	Keyhole neurosurgery	The risk card and the insertion trajectory safety zone sleeve are generated and update in real time, to provide visual and quantitative feedbacks for manual planning	Provide an assistive tool	Retrospective (N = 8)	Vessels, and the ventricular system	Preoperative CT/MRI scans.	Automatically generated paths were 2.6 mm further from blood vessels compared to manually planned paths	Brain shift was not considered	University of Jerusalem
Zelmann et al. [13], 2013	Epilepsy— Mesial temporal lobe implantations	Cost function and reward function optimization	Increase recording coverage of the target volume by estimating the EEG recorded from each contact	Retrospective 27 trajectories in 11 hemispheres	Blood vessels, sulci, lateral ventricle, and other electrodes	T1-weighted with and without gadolinium	25/27 trajectories (92.5%) satisfied the proposed surgical constraints	Wilcoxon test was used for statistical comparison	Montreal Neurological Institute
Momi et al. [22], 2013; [30], 2014; ([48], 2017)	Epilepsy	Multi-objective cost function optimization	Safe trajectories	Retrospective N = 3, T = 26 (Animal study)	Lateral ventricles, thalamus, cerebellum,	MR images	Straight tool		Politecnico di Milano
Zelmann et al. [31], 2015	Epilepsy	Global optimization	Safe paths; maximizes the recording volume,	Retrospective (N = 20)	Sulci, Ventricles, Reducing muscle crossing, ear canal and surrounding cartilages	MRI for targets; CT for skull; CT angiography for vessels	Straight tool, Median volume coverage of 419 mm^3^ versus 23 mm^3^ for MP	The target is a region and not a point Plan for a set of 3 electrodes at a time	McGill University
Hamzé et al. [57], 2015	DBS	Geometric constraint solving plus using FEM for brain shift simulation	Using anticipation of brain shift for path planning	Retrospective N = 1	Blood vessels were difficult to segment from MR images so sulci were considered; ventricles	MRI	Straight tool, More than 50% shrinking was anticipated considering a possible brain shift.	The main cause of brain shift is a loss of Cerebro-Spinal Fluid (CSF) surrounding the brain.	University of Strasbourg
Trope et al. [29], 2015	Neurosurgery	In the automatic approach, skull surface was evenly sampled with ~1000 points, each considered as a candidate entry point and costs were estimated	Three methods were compared. (1) conventional; (2) assistive (visualization), and (3) automatic	User study Five neurosurgeons	Ventricles, blood vessels, high-density white matter fibers tractography, and the cortical functional motor, visual, sensory, and speech areas	Multi-sequence MRI studies: (1) T1-weighted gadolinium-enhanced images, (2) FLAIR, DTI, and (3) fMRI	Two computer-assisted surgical path-planning approach are superior to manual path planning		University of Jerusalem
Sparks et al. [32], 2016	Epilepsy	Depth-first search TheEpiNav was used for manual plan assessment	Lower risk, Increased grey matter sampling, Shorter length, and Surgically preferred entry angles	Retrospective (N = 18, T = 165)	arteries, veins, sulci, conflicts between electrodes	CT angiography, 3D phase contrast MRI	Straight tool, Planned 12 electrodes within 1 min, compared to 2–3 h for MP	Arteries, Veins, and Sulci are hard constraints, Grey Matter to White Matter Ratio is a soft constraint	University College London
Hamze et al. [58], 2016 [56], 2016	DBS	Multi-objective optimization method based on (Non-dominated Sorting Genetic Algorithm (NSGA)	Compare the proposed method with weighted-based methods (WS)	Retrospective N = 10	Cortical sulci in which the vessels usually are located, and ventricles	MRI	Straight tool, Approximately 37% of the solutions found by NSGA-II could not be found by WS exploration.	Most of the current automatic path planners use single objective optimization	University of Strasbourg
Sparks et al. [33], 2017	Epilep0sy	Anatomy-driven multiple trajectory planning Graph search and cost function optimization Discard trajectories that are very close to each other	Target planning as well as trajectory planning	Retrospective (N = 20, T = 190)	Arteries, Veins, Sulci	3D T1-weighted MPRAGE scan MR angiography (MRA) and venography (MRV)	Lowered the quantitative risk score in 83% of electrodes; Found suitable trajectories for 70% of electrodes Trajectory suitability for ADMTP was 95% if traversing sulci not included in safety criteria. Computing between 7 and 12 trajectories in 54.5 s	The manual plans were determined by the consensus of two neurosurgeons A two-tailed Student’s *t* test was used to evaluate the trajectories and where the null hypothesis was that the methods return similar values.	University College London
Scorza et al. [66], 2017	Epilepsy	Exhaustive (brute force) Search-based optimization	Trajectories optimized for vessel distance and insertion angle	Retrospective N = 20	vessels and sulcal entry	CT angiography volume CT acquisition of head bone or, alternatively, a T1-MRI	Straight tool, Improved 98% of the cases	Initially, the user defines a set of EP and TP to represent the desired intracerebral investigation strategy.	Vicomtech-IK4
León-Cuevas et al. [18], 2017 ([59], 2015)	Keyhole neurosurgery	Search-based method (Using fuzzy logic for risk evaluation)	Safe and short trajectory			CT/MRI	Straight tool	Voxel-based cost map Risk evaluation using fuzzy logic	
Vakharia [47], 2018	Epilepsy ablation phase Trajectory planning for hippocampal laser trajectories	EpiNav software	Reach to expected ablation volumes and safety	Retrospective (N = 25)	Vasculature or Sulci (in cases where vascular segmentation not possible).	T1-weighted MRI scan and CT	Planner results in significantly greater ablation of the amygdala and amygdalohippocampal complex and less residual unablated mesial hippocampal head , and reduced ablation of the parahippocampal.	This study is underpowered It is estimated that ~250 patients would need to be enrolled to detect an increase seizure freedom rate of 20% with a power of 90% at a significance level of *p* = 0.05.	University College London
Dergachyova et al. [12], 2018	Deep brain stimulation (DBS)	Multi-objective optimization	Optimize trajectory and target point	Retrospective study (N = 18)	Sulci mesh; anatomical structures;		Improvement in the expected outcome	Used Anatomo-clinical atlases for target (stimulation) point optimization Volume of tissue activated	Université de Strasbourg
Li et al. [50], 2019	Laser interstitial thermal therapy	Used machine learning approaches (random forest and linear regression) to predict entry and target points	Maximizing the ablation of the amygdalohippocampal Complex (AHC)	Retrospective N = 10	Sulci and intracranial vasculature, Brainstem, Ventricle	MRI T1 MPRAGE; synthetic CT (pseudo-CT),	Straight tool, Both linear regression and random forest approaches showed similar results.	Maximal ablation of the mesial hippocampal head and amygdalohippocampal complex improves seizure freedom rates	Department of Neurosurgery, Tangdu Hospital
Vakharia et al. [20], 2019	Epilepsy	EpiNavTM Software		Prospective study (N = 13)		Digital subtraction catheter angiography (DSCA)	30% lower risk scores		University College London
Vakharia et al. [49], 2019	Laser interstitial thermal therapy	Used EpiNav platform	Multi-center study to compare MP and CAP	Retrospective N = 95	The lateral ventricle: no-entry zone Vasculature, brainstem, and sulci: critical structures with 3 mm safety margin	T1 image is used to generate a patient-specific whole-brain parcellation and pseudoCT [73]	Straight tool	Blinded external expert raters were significantly more likely to prefer automated to manually planned trajectories	UCL Institute of Neurology, National Hospital for Neurology and Neurosurgery, London
Vakharia et al. [66], 2019	Epilepsy	Used EpiNav platform	Comparing MP with automatically generated path	Retrospective (N = 13, T = 116)	Blood vessel or sulcus, and ventricles	T1- weighted gadolinium-enhanced images; 3D FLAIR scans	Straight tool. Significant improvement in electrode length, drilling angle, gray matter-sampling ratio, minimum distance from segmented vasculature	The user defines target points as ROIs for electrode sampling Safety margin: 3 mm	National Hospital for Neurology and Neurosurgery, London
Vakharia et al. [65], 2020	Anterior two-thirds corpus callosotomy	EpiNav platform was used	Feasibility study with probabilistic tractography validation	Retrospective N = 10	Vessel, ventricular system, Cingulate gyri, and the fornix	Isotropic 3D-T1 magnetization-prepared rapid acquisition with gradient echo (MPRAGE)	Less residual interhemispheric connectivity	Vascular segmentations derived from DSA. They acknowledge that DSA is not routinely acquired for LITT procedures	University College London, London
Marcus et al. [64], 2020	Stereotactic Brain Biopsy	The SurgiNav platform was used	A retrospective comparative pilot study	Retrospective N = 15	Sulci and ventricles	T1-weighted, gadolinium-enhanced MRI	Trajectories were more perpendicular to the skull and	Feasible trajectories	National Hospital for Neurology and Neurosurgery, London
Wankhede et al. [45], 2022	Neurosurgery	Brute force search	Maximize hippocampi ablation	Retrospective N = 6	Blood vessels, Sulci, and ventricles	T1 weighted MRI with and without contrast agent	Straight tool The average penetration ratio 88.13 ± 23.23%		Children’s National Medical Center, Washington DC
Cai et al. [74], 2022	DBS	pre-operative DBS path planning	Implement a coarse-to-refine deep learning segmentation method						Institute of Intelligent Machines, HFIPS, Chinese Academy of Sciences
Marusich et al. [75], 2023	DBS	[17,23]	Software introduction	N = 1	cortical sulci, eloquent cortical regions, and ventricles and surrounding cognitive regions	(3T and 7T) MRI		Providing surgeons with quantitative, patient-specific vasculature data	Stanford University, Stanford
Pantovic et al. [5], 2024	DBS	Deep reinforcement learning (DRL)	Compare DRL with conventional techniques	Retrospective 18 + 13 patients A total of 55 trajectories for training and validation	Ventricles, cortical sulci, and vessels.	MR images and postoperative CT scans	2.3% improvement in average accuracy in proximity to critical structures and 19.4% improvement in average orientation angle	Computation times increased from 2 to 18 min.	University of Strasbourg

Hong et al. [76] developed a path-planning method for Deep Brain Stimulation (DBS) procedures using a steerable flexible needle. They developed an RRT* (a variant of RRT that provides a more optimal path [77]) path planner technique and tested it in simulation on a realistic 3D model of the brain considering these constraints: (1) avoid penetration to the midline, (2) limit the entry point to posterior to the hairline, (3) sulci, (4) ventricles, and (5) large blood vessels. A multi-variable cost function was used to consider the length of the path and distance from vessels to generate a path. They compared the path-planning flexibility when using a steerable needle versus a straight needle and concluded that path planning using a steerable needle is superior for the following reasons: (1) more flexibility with entry points, and (2) generates safer paths.

Segato et al. [78] used an RRT*-based path-planning algorithm for DBS intervention in the context of EDEN2020. Their path planner initially outputs a set of piece-wise linear feasible paths without considering the curvature constraints. In this study, they considered white matter, fiber tracts, and deep gray matter nuclei as critical regions to avoid potential damage to pivotal functions—in addition to other critical structures. Then, they use an evolutionary optimization technique to smoothen the paths, reduce their lengths, and optimize the insertion angle. This is done using the vertexes of each piece-wise linear segment of the generated feasible paths to form a point set required to create Non-Uniform Rational Beta Splines (NURBS). Finally, an exhaustive search is conducted to select the best path among all the feasible paths. 10 healthy volunteers have been enrolled in this path-planning study which is only a computational study. The rectilinear stereotactic trajectories (RTs) manually defined by surgeons, were compared with curvilinear trajectories automatically computed.

Batch Informed Tree (BIT*)—The BIT* algorithm balances the benefits of a graph search and sampling-based approaches. BIT* solves continuous path-planning problems by alternating between random sampling and heuristic search to approximate the search space and search for a solution. Once the first feasible path is found, the search space is confined within an ellipsoidal region. Although this technique is similar to informed-RRT* (a variant of RRT*) in using the ellipsoidal region to confine the search space, the BIT* outperforms informed-RRT* in terms of computation time. Favaro et al. [79] developed a BIT* path planner for neurosurgery applications using the EDEN2020 steerable tool. They compared their proposed path planner with several variants of RRT* in simulation. They also conducted a study to compare the standard straight trajectory-planning method and their novel curvilinear planner integrated with the EDEN2020 programmable bevel-tip needle.

B. Adaptive Hermite Fractal Tree (AHFT)

The same team from Imperial College later developed a new parallelizable path-planning approach called Adaptive Hermite Fractal Tree (AHFT) that generates a three-dimensional (3D) path while addressing the curvature (kinematic) constraints given the start and goal points. Using a preoperative neurosurgical simulated environment, they showed that their method is robust to the target position and heading perturbations due to brain shift with burr hole as well as loco-regional brain tissue distortion with the steerable electrode going through brain tissue. The main purpose of this project is to develop a technology for Convection Enhanced drug Delivery (CED) using a bio-inspired steerable needle, i.e., EDEN2020 [80,81]. The RRT-based algorithms are incompatible with the GPU requirement, due to single instruction multiple data operations, which limits scalability to multiple CPUs with limited computation power [82]. In comparison, AFTs can run on the GPU allowing real-time path planning. Based on one study [72], RRT’s success rate is 42% less than an AFT in finding solutions for complex critical structures. 

The simulation results showed that this technique takes an average of 0.22 s to produce at least one AFT path 94.23% of the time, while the branch length is 20 mm and maximum curvature is 17 mm.

C. Optimization-Based Approaches

The path-planning problem can be considered an optimization problem. To develop a path planner for STING needle, Ko et al. [67] proposed a gradient-based optimization method. They defined the path as a polynomial curve and optimized the coefficients based on the constraints and the critical structures.

Based on previous works by Flaßkamp et al. [83] and Hackenberg et al. [54], Sauerteig et al. [84] improved the mechanical system model for the cannula accounting for several important aspects including the electrostatic interaction among concentric tubes that resulted in a more accurate model that is essential for the realization of the cannula in future experiments. They used numerical optimization methods through the Python toolbox CasADi developed by Andersson et al. [85] to evaluate the system in simulations. They modeled the critical structures as ellipsoids and automatically generated paths by minimizing two cost functions: (1) arc length and (2) distance to the target set. They then developed a new path-planning algorithm [86] via a homotopy on the obstacle positions, similar to ideas presented [87,88] for unmanned aerial vehicles. Using the homotopy approach, they were able to resolve some of the issues encountered by their previous approach, as the solver could not converge when dealing with thousands of ellipsoidal obstacles.

D. Reinforcement Learning-based Approach

Kumar et al. [89] developed a reinforcement learning-based approach for path planning for flexible needle insertions. The goal was to generate a Bezier curve that safely reaches out to the goal point. Control points were generated by a reward-based reinforcement technique. They compared the path generated using their approach with the traditional sampling-based algorithm such as RRT*, through simulation and reported that their method generated smoother and shorter trajectories compared to RRT*. Dundar et al. [90] used the Q-learning algorithm, which is a model-free reinforcement learning algorithm. It was run on a labeled voxel set to list the cortico-tumoral paths that could result in the maximum tumor tissue removal while minimizing the collateral damage to the sounding tissues and critical structures. Table 4 summarizes publications related to steerable needle trajectory planning.

#### 4.2.2. Multi-Target Trajectory Planning for Steerable Needles

Steerable needles exhibit complex, non-linear kinematics, making accurate modeling and control difficult. Predicting the precise path of these needles in heterogeneous tissue environments is challenging as interaction between the needle and the tissue can cause unpredictable deformations, which complicates path planning and requires real-time adjustments. Similarly, multi-target path planning for steerable needles requires optimizing multiple objectives, such as minimizing tissue damage, avoiding critical structures such as blood vessels and nerves, and ensuring accurate localization of targets [91,92] while taking into account the tissue deformation. This multi-objective optimization is computationally intensive. The need for real-time path adjustment based on dynamic changes in tissue properties and elastic deformation detected by real-time imaging [93] adds to the computational burden and requires sophisticated control algorithms. Additionally, pre-operative path planning requires sophisticated and precise tissue modeling [94] to predict the deformation caused by interactions between the tissue and multiple needles. This especially is very challenging, when the goal is to place all needles simultaneously or to use a single needle and partially retract the needle before aiming for a new target [91]. In scenarios where the goal is to place, treat, and fully retract the needle before proceeding with the next target, single trajectory-planning techniques, as covered in the previous section, could be employed. Currently, steerable needles are not utilized in clinical settings for either single-targeting or multi-targeting scenarios. Audette et al. [11] and Babaiasl et al. [95] have published survey papers specifically on steerable needle technologies, discussing potential clinical applications and technical challenges. However, they have not reported any clinical trials in any medical specialty. Therefore, these considerations for automatic path planning for multi-target scenarios are still in the development phase and pertain to future possibilities in medical procedures.

### 4.3. Path Planning for Concentric Tube Robots

**Figure 9:**
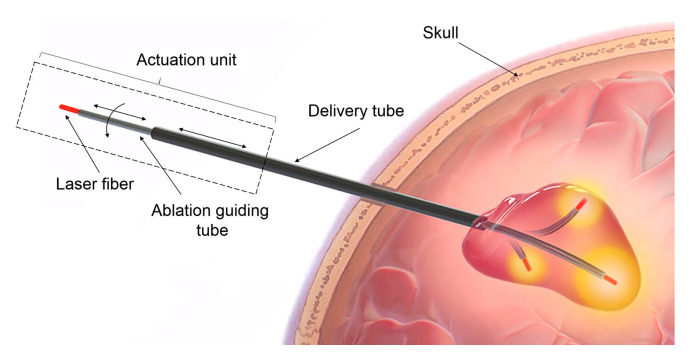
CTR is composed of an outer delivery tube and an inner ablation guiding tube housing the laser fiber (red) within its inner lumen. The laser fiber deposits energy (yellow) in a spherical region. Reprinted/adapted with permission from Ref. [101].

**Figure 10:**
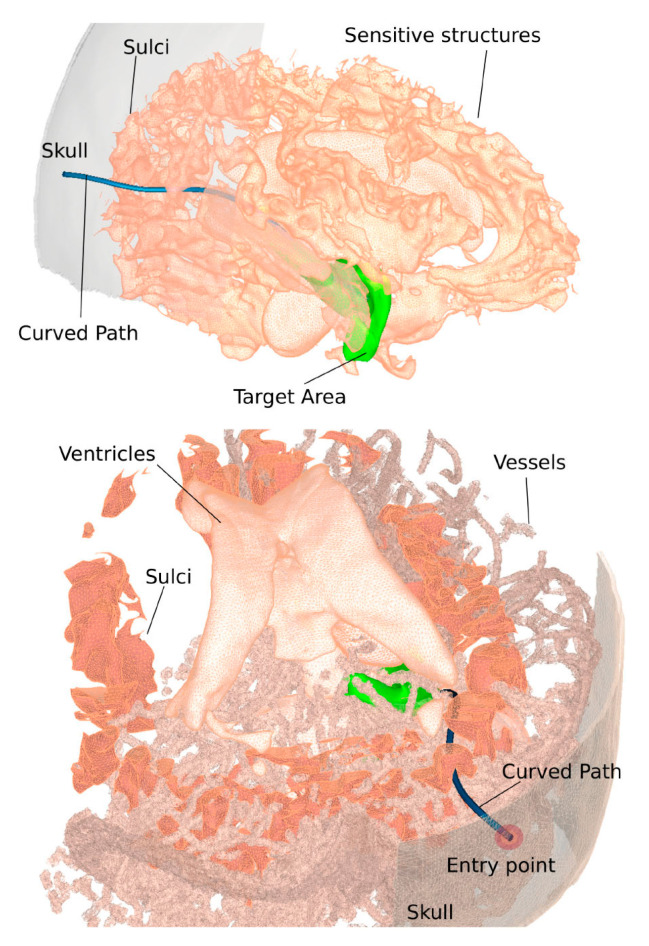
The optimal collision-free path is shown. Reprinted/adapted with permission from Ref. [69].

A concentric tube robot (CTR) can provide needle guidance and allow the different targets within the body to be reached through natural orifices or single incision [96]. CTRs are comprised of a series of pre-curved and super elastic tubes with different diameters that are concentrically assembled. The combination of these tubes can be telescopically extended or retracted and can be rotated both clockwise and counterclockwise, providing a full tip-pose control [97]. For path planning of CTR needles, unstable CTR needle configurations should be avoided in addition to the anatomical critical structures and kinematic constraints of the needle [97]. Figure 9 shows a concentric tube robot developed for laser ablation [69]. Figure 10 shows an example optimal collision-free path generated for a concentric tube robot. 

#### 4.3.1. Single Trajectory Planning

A. Sampling-based techniques

*Probabilistic Road Map*—Leibrandt et al. [97] used the Probabilistic Road Map (PRM) technique to generate an undirected graph with each vertices representing a random, stable, and collision-free needle configuration. An edge connecting two vertices represents a possible transition from one configuration to another. A* graph search algorithm was used to search for the optimal trajectory within the limits of the generated PRM, so the needle can be moved from the current configuration to the desired configuration. The Euclidean norm between two configurations (two vertices) is considered an admissible heuristic function for the A* algorithm. Their path planner required 3 to 5 min of preoperative precomputation, and 1 to 10 s of intraoperative computation time [97].

RRT*—Bergeles et al. [98] proposed using RRT* path-planning technique for concentric tube robots that allows the utilization of generally unstable CTRs by generating a trajectory that ensures the robot operates in its stable configuration workspace, while avoiding critical structures and adhering to kinematic constraints. As a result, they concluded that there’s no need to design tube sets for global stability. Regardless of what type of tools are used for minimally invasive neurosurgical interventions, common challenges should be addressed for successful path planning. For instance, different sources of uncertainty should be considered during the path-planning process. 

In addition, critical structure segmentation error, patient-to-image registration error, brain shift during surgery, and in the case of robotic intervention, robot to scanner registration error, and mechanical uncertainty are the main criteria that should be considered during the path planning. These sources of uncertainties are handled primarily by adding a ‘safety margin’ (typically 2–3 mm) around the critical structures to avoid penetration through these structures. However, Frisken et al. [99] handled the uncertainties due to segmentation and brain shift more rigorously. They used entropy as a reliable quantitative measure of uncertainty for the accuracy of image segmentation. They generated patient-specific probabilistic segmented uncertainty zones based on image entropy, where entropy correlates to each voxel’s density function. On the other hand, they used finite element analysis to predict the brain shift after making a burr hole in the skull. Then they combined these uncertainties into a single risk map. 

In the context of path planning, some techniques rely on neurosurgeons’ knowledge to generate admissible paths. Corbetta et al. [100] developed a path-planning algorithm based on answer set programming to translate the requirements and the experts’ knowledge into the objectives of the optimization procedure. This also allows flexibility to change the optimization requirements based on the requirements of each clinical case.

B. Optimization-based methods

For concentric tube robot path planning using a multi-objective particle swarm optimization algorithm refer to [101,102]. Table 5 summarizes publications related to concentric robot trajectory planning.

**Table 4:** Summary of publications on steerable needle path planning.

Authors/Year	Application	Path-Planning Method	Objectives	Evaluation Method	Critical Structures	Imaging Method	Tool Type/ Accuracy/ Efficiency	Assumptions/Evaluation Measures/Other Descriptions	Group/ University Name
Caborni et al. [72,103], 2012	Neurosurgical Intervention	Reachability Guided Rapidly exploring Random Trees (RG-RRT)	Minimizes the length of the path, the distance from vessels, and nervous bundles, also minimize accumulated risk along the trajectory	Phantom study	Vessels and nervous bundles	MRI image	Soft tissue intervention and neurosurgical guide (STING)	RG-RRT improves the sensitivity to the distance metric while expanding the tree compared to RRT	Politecnico di Milano
Bano et al. [68], 2012	Neurosurgical Intervention	Path optimization	Path planning for a biologically inspired	Simulation	Obstacles		Shorter paths	Minimize damage to the tissue	Imperial College, London
Young Ko et al. [67], 2013	Keyhole Neurosurgery	A gradient-based optimization using a curvature polynomial is adopted	A novel path planner that satisfies constraints on both the maximum curvature of the final trajectory and its derivative.	Simulation	Obstacles		Soft tissue intervention and neurosurgical guide (STING)	A 4-mm two-part prototype needle was used Minimum radius of curvature of ~70 mm	Imperial College London
Liu et al. [81], 2015	Surgery	Parallel real-time path planning based on moduli space-planning algorithm	To develop and validate a real-time path planner	Simulation	Obstacles	Their method works directly with raw MRI image data in the voxel format that simplifies image processing and boosts system performance	Steerable needle	The path planner updates the path based on the brain deformation in real time to track the moving target	Imperial College London
Liu, et al. [71], 2016	Keyhole Neurosurgery	Adaptive fractal trees (AFT),	GPU accelerated parallel planner for real-time path planning	Simulation	Obstacles	CT images	EDEN2020* programmable bevel-tip needle AFT is capable of addressing nonholonomic constraints due to needle geometry and function; AFT performs better than RRT	A common issue with most of existing path-planning methods is that they are based on sequential search, relying on serial CPU computing	Imperial College London
Favaro [53], 2017	Target point within the white matter of a sheep’s brain	Exhaustive search	Short computational time and good capability in segmenting gyri and sulci	Animal study (Sheep)	vessels and risky structures.	T1w images	Catheter	Danielsson distance map filter provided in the ITK library was used	Politecnico di Milano, Milan
Favaro et al. [79], 2018	DBS	Batch informed Trees—a Sampling-based planner	Comparison of standard rectilinear trajectory planning against this novel curvilinear trajectory planner	Data from one healthy volunteer	Blood vessel, thalamus, and ventricles	3D T1-weighted MRI and 3D high-resolution time-of-flight	Programmable bevel-tip needle from EDEN2020 program	Vessels are more likely located at the bottom of sulci	Politecnico di Milano
Segato et al. [78], 2019; [104], 2021	DBS	RRT*-Based Raw Planning, optimization then Exhaustive Search for Best Path	To avoid Fiber Tracts and Deep Gray Matter Nuclei Curvilinear trajectories were compared to rectilinear stereotactic trajectories in terms of efficacy and safety	10 healthy controls and 10 cases	Motor fibers, deep gray matter nuclei, vessels	T1 and TOF-MRA images, Diffusion MR tractography	Curvilinear tool developed for EU’s Horizon EDEN2020 project; Curvilinear trajectories provided safer trajectories	There is a tradeoff between the an optima path generated by graph search-based approaches and path approximated using sampling-based methods.	Queen Mary University of London
Hong et al. [76], 2019	Neurosurgery	RRT	Minimize tissue damage while allowing for a much greater selection of entry points	Simulation	Neural structures and Vessels				Multi-Scale Robotics Laboratory, ETH Zürich
Hackenberg et al. [54], 2021	Tumor ablation	TransWORHP software		Retrospective N = 1	Blood vessels or cerebral sulci	They used MeVisLab [105] for MRI image processing	Curved cannulae	Hysteresis effect scan damage the brain	University of Bremen
Pinzi et al. [69,80], 2019, and 2021	Laser interstitial thermal therapy	GPU-accelerated computer-assisted planning based on adaptive fractal tree and adaptive hérmite fractal tree method	The clinical feasibility and potential of curved LiTT trajectories through steerable needles were investigated	Retrospective N = 5	Avoid vasculature, sulci, brainstem, and the ventricular ependyma (safety margin = 7.5 mm) Minimize ablation of parahippocampal gyrus and surrounding critical structures	T1 MPRAGE and synthetic CT (pseudo-CT)	programmable bevel tip needle (from EDEN2020), significant improvement in ablation zone and risk score reduction	In some cases, more than one path is required too fully ablate the tumor using straight tool	Imperial College London
Corbetta et al. [100], 2021	Keyhole Neurosurgery	Answer Set Programming	To translate the knowledge from experts into the objective function for the path optimization	Simulation	Arterial blood vessels, and ventricles	(1) Time-of-Flight (ToF) MRI for vessels, (2) T1 for brain cortex, skull surface, arterial blood vessels, ventricles and deep grey matter	EDEN2020 programmable bevel-tip needle The proposed approach is superior to manual path planning in terms of cost and total path lengths	Most of current path-planning methods lack the flexibility to change and adaption in real time intraoperatively	Politecnico di Milano
Kumar et al. [89], 2022	Keyhole Neurosurgery	Reinforcement learning-based method to generate kinematically feasible trajectories	The proposed approach was compared with a sampling-based RRT* path planner	166 datasets were used; 29 samples for training datasets: 29 Testing datasets: 137	Ventricles and vessels	MR angiography, MRI-T1	Flexible Needle	Control points of the Bezier curve are generated using a reward-based reinforcement learning method	Indian Institute of Technology Madras
Frisken et al. [99], 2022	Epilepsy	Uses probabilistic approach to create the risk (cost) map	Incorporating Uncertainty into Path Planning	They used a commercial dataset of 62 MRI scans that were segmented by a trained neuroradiologist	Ventricles and blood vessels that lie within the sulcal folds of the cortical surface of the brain	MRI They used Z-scores to normalize images, then cropped and sub-sampled image by a factor of two to improve image processing efficiency	Continuum robot; They used a hybrid approach for path planning: A straight canula from the brain surface to hippocampus through which a tiny continuum robot is Inserted to follow the curve of target region	Segmentation uncertainty and uncertainty due to brain shift were considered as a main source of uncertainty	Brigham and Women’s Hospital, Boston
Dundar et al. [90], 2022	Keyhole Neurosurgery	A model-free (Q-learning algorithm) reinforcement	To find the best linear and nonlinear surgical path	Retrospective N = 1	Fiber tracts, arterial, venous vessels, and basal ganglia, ventricular, and thalamus	T1-weighted, T2-weighted, FLAIR	Linear and nonlinear tools	Reinforcement learning algorithm was run on labeled voxels generated by heuristic-based path planner to find path with maximum ablation while avoiding damage to functional anatomical tissues	Bezmiâlem Vakif Üniversitesi

**Table 5:** Summary of publications on concentric tube robot path planning.

Authors/Year	Application	Path-Planning Method	Objectives	Evaluation Method	Critical Structures	Imaging Method	Tool Type/ Accuracy/ Efficiency	Assumptions/Evaluation Measures/Other Descriptions	Group/ University Name
Bergeles et al. [98], 2013	Keyhole Neurosurgery	RRT*	Consideration of stability in robot design and path planning was novel aspect of this paper	Retrospective N = 1	Not specified	MRI or CT	Concentric tube robotic	Tube sets does not need to be designed to be globally stable	Boston Children’s Hospital
Granna et al. [101], 2019 ([102], 2017)	Laser-induced thermotherapy	Multi-objective Particle swarm optimization	Both task-specific planning and robot-specific planning were conducted	Retrospective N = 3 (N = 15)		MRI	Concentric tube robotic	Non-homogenous convection of ablative energy, for instance due to heat sinks (vessels, cysts or the ventricular system) or convection barriers through carbonization in multiple overlapping areas were not considered	Leibniz Universität
Flaßkamp et al. [83], 2019	Keyhole Neurosurgery	Defining path using differential equations	To find a feasible path and minimize damage to brain due to issue with follow-the-leader behavior	Simulation	Obstacles with geometrical shape	MRI	Concentric tube robotic		University of Bremen
Sauerteig et al. [84], 2022	Keyhole Neurosurgery	Defining path using differential equations	Increase access using curvilinear trajectories	Simulation	Vessels, and furrows (sulcus)	MRI	Concentric tube robotic This tool enables accessing part of the brain that is not accessible by straight tool	Electrostatic model was considered for the tool	Technische Universit¨at Ilmenau
Hoffmann et al. [86], 2023	Keyhole Neurosurgery	CasADi by Andersson et al. [85] was used for the optimization	To develop an easily solvable optimization method	Simulation on real-world data obtained from labeled MRI scans	Ellipsoidal obstacles	MRI	Concentric tube robotic	The concept of homotropy was used on some of the obstacles, meaning that they were removed and added after the initial guess followed by path adjustment.	Technische Universit, Germany
Pehlivanoğlu et al. [70], 2023	Neurosurgery	Dijkstra, A*, and their aggressive variants	Consider all areas of the brain and for path-planning software that is independent of the tool type	Simulation	Vessels and corticospinal tracts	MRI and DTI	Curvilinear surgical paths	No non-holonomic constraints are considered for the surgical tools	Kocaeli University

#### 4.3.2. Multi-Target Trajectory Planning for Concentric Tube Robots

Similar to steerable needle technologies and their applications for multi-target scenarios, concentric tube robots are not currently used in clinical settings for either single-targeting or multi-targeting procedures. The technical challenges are similar, but concentric tube robots face additional difficulties. These include increased complexity in their kinematics, greater sensitivity to manufacturing tolerances, hysteresis effects, and more intricate control requirements due to their flexible, nested tube structure. Designing pre-formed arcs for concentric tube robots adds another layer of complexity, as it requires precise modeling and fabrication to ensure accurate and predictable movements.

Furthermore, the interaction between multiple concentric tubes and the surrounding tissue can lead to compounded deformations, making precise path planning and real-time adjustments even more challenging. Alfalahi et al. [106] have discussed potential applications for concentric tube robotic systems. However, they have reported no clinical trials of these technologies, either for single-target or multi-target scenarios.

Mitros et al. [107] have reported progress on concentric tube development robots and different clinical applications. However, they have not reported any human studies using these technologies. It should be noted that Virtuoso Surgical (virtuososurgical.net) and EndoTheia, Inc. (www.endotheia.com) are two startup companies originating from the Medical Engineering and Discovery Lab (MEDLab) at Vanderbilt University. Virtuoso Surgical aims to bring concentric tube robots to operating rooms, while EndoTheia focuses on commercializing steerable sheaths for flexible endoscopy. Therefore, developing automatic path-planning algorithms for these systems could be highly valuable in the future, potentially enhancing their effectiveness and facilitating their adoption in clinical settings, primarily for single-target scenarios and potentially for multi-target scenarios as well.

## 5. Discussion

### 5.1. Current Progress in the Field

Different search-based, sampling-based, potential field-based, and AI-based path-planning algorithms have been developed and retrospectively have been tested and evaluated. Most of these studies have confirmed that neurosurgeons found the automatic path-planning algorithms better or equally acceptable compared to their manual path-planning techniques. Most of these automatic path-planning algorithms rely on semi-automatic or manual segmentation of brain structures as some of the brain structures such as the skull, and ventricles can be automatically and easily segmented with a low computation time. However, structures such as vessels and hippocampus require some level of manual segmentation or manual adjustment. Therefore, the overall time for path planning using an automatic path-planning algorithm takes hours rather than minutes to complete. This still is an improvement as the semi-autonomous segmentation could be done by someone other than neurosurgeons, freeing their time for more clinical practice. It should be noted that neurosurgeons still need to review the segmentations and confirm the quality of the segmentations that are going to be used by automatic path planner for generating safe paths. 

Reducing the segmentation time and fully automating the segmentation requires high-quality images to identify different brain structures and improve image contrast. Higher contrast images will improve the accuracy of automatic image segmentation algorithms and will improve the overall path-planning time. Some research groups have used ultra-high field strength MRI scanners [75] to overcome this issue. Other groups proposed optimized MR imaging sequences such as susceptibility-weighted imaging (SWI) to improve the visualization of the vessels [108,109,110,111]. SWI is superior to T1-weighted images in the visualization of small blood vessels [112]. T1-weighted images enhanced by contrast agent administration mainly visualize arterial vessels. However, SWI is a relatively new MR imaging sequence for improved visualization and can visualize brain blood vessels with diameters less than a millimeter [113]. 

**Figure 11:**
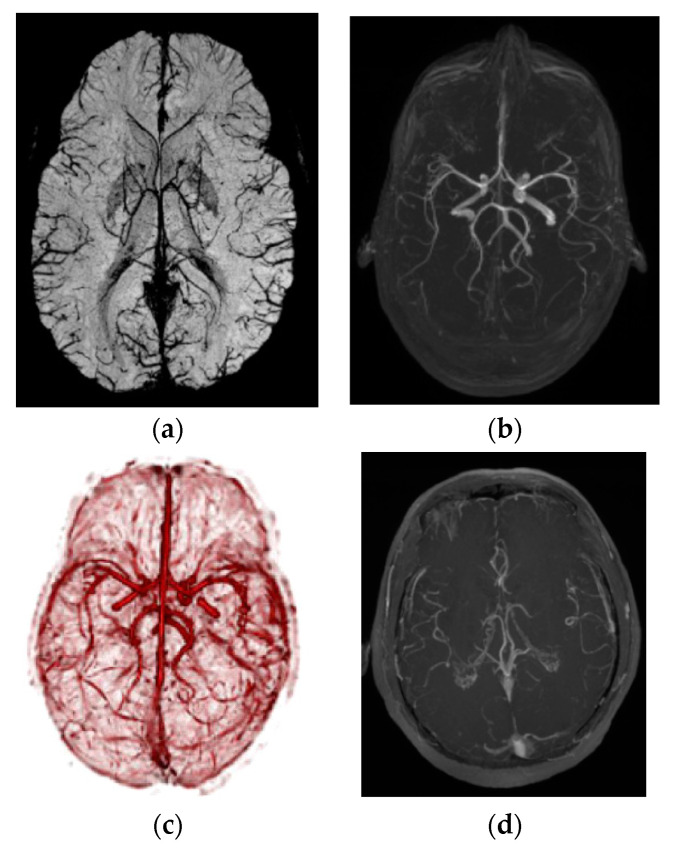
(**a**) SWI, (**b**) TOF, and (**c**) 3D rendering of combined SWI-TOF dataset, and (**d**) T1w with gadolinium. “Reprinted/adapted with permission from Ref. [24].

Mahvash et al. compared the image quality of SWI and stereotactic contrast-enhanced T1-weighted images for deep brain stimulation applications. They concluded that SWI could visualize, on average, 2.4 additional vessels, (range 1–4 vessels), within the region of interest in all patients (N = 33) not visible using stereotactic contrast-enhanced T1-weighted images. More importantly, mesencephalic vessels at the endpoint of electrodes, which can cause fatal hemorrhages, are reliably visualized with SWI [112]. Figure 11 shows different imaging protocols and imaging quality to identify vessels. Future developments are necessary to improve vessel visualization. In addition to path-planning algorithms that have been developed for intervention, different path-planning software were developed for training purposes, such as a virtual reality tool called VirSSPA [114,115]. VirSSPA allows surgical planning optimization, path-planning time reduction, and improves operative results. In the future, more advanced and up to date path planner simulators are required to employ the current state-of-the-art technologies that will allow the neurosurgeons to interactively work with the software to generate the best and safest path with greater time efficiency. In the following subsections, we will highlight some of the open research challenges and future directions.

### 5.2. Open Research Challenges 

*Clinical adoption*—Despite the significant efforts of many research groups in developing automatic path-planning tools, and the clear advantages of such systems in enhancing precision and assisting neurosurgeons to plan faster and more efficiently, the clinical translation of this technology has been very slow. Some of the automatic path-planning software systems, such as Computer-Assisted Path-planning Software (CAPS) [116], were developed a decade ago, but have not been widely adopted. Our survey also illustrates that there have been very limited number of prospective studies to validate these technologies. Regulatory pathways to obtain approval for such studies and the risk associated with these types of studies might be one reason behind the slow adoption and limited prospective human studies. Benchmark studies are required to improve the rate of adoption of these systems. Other potential drawbacks toward wide clinical adoption of these systems should be identified to create a roadmap for future endeavors.

*Real-time Path Planning*—The automatic path-planning algorithms, especially those developed to run on GPU, are extremely fast. It should be noted that segmenting the critical structures and the target regions is a critical step toward automating the path-planning process. The brain structure segmentations are performed either manually, semi-automatically, or automatically. Fully automated and precise segmentations are still not feasible for certain structures such as vessels. On the other hand, manual segmentation or modification is very time extensive. Therefore, the overall time for automatic path planning could be considerable due to the image segmentation aspect of the process.

Real-time path planning is more critical for intraoperative path planning and path adjustment due to brain shift. This requires faster path-planning algorithms on the one hand and improved real-time imaging of brain structures, especially blood vessels on the other hand. High quality imaging is the key to fully automatic and quick segmentation of the brain structures to inform the automatic path planner. Different approaches have been developed and tested for automatic segmentation including coarse-to-refine neural network models [74]. It is known that there are several gaps and shortcomings in the automatic segmentation of different brain structures. As such, further developments are required to automate the segmentation task. Discussing various segmentation techniques and tools is out of the scope of this paper; for more information refer to [117]. 

*Integration with Robotic Systems*—These novel automatic path-planning algorithms could be integrated with MRI- or CT-compatible robots for image-guided autonomous interventions. Integration with MRI-compatible robots will enable real-time image guidance, improving the precision and safety of interventions. Robotic systems, such as the Neuromate system (Renishaw Mayfield SA), already exist and can automatically align the intervention tool holder along the automatically generated path [103]. However, these systems cannot operate within the bore of an MRI scanner which could provide better imaging guidance. Further developments in robotic systems are needed to enable real-time image-guided in-bore interventions in the future. Open-source software plays a crucial role in achieving this goal. Automatic path-planning algorithms developed on top of open-source visualization platforms such as 3D Slicer [118,119,120] and Tactics [121], and their integration with the Robot Operating System (ROS) as demonstrated in [104], could further streamline the integration of image-guided robotic systems with these automated path-planning tools. This approach would also provide better access to the community, facilitating wider adoption.

### 5.3. Future Directions

In addition to the challenges listed in the previous subsection that can guide future research directions, there are other topics that could be investigated by the research community as follows:

*Long-term Outcome Studies*—Vakharia et al. [73] reported on the most recent advance in the field up until 2020 and compared different path planners. Based on this paper, future developments within epilepsy surgery include trajectory planning for automated laser interstitial thermal therapy and machine learning algorithms to improve generalizability. In addition to investigating the optimality of the path generated using different path-planning algorithms compared to manual path planning, the next step is to determine if this approach results in improved seizure-free outcomes and reduced neuropsychological morbidity [50]. The research community should prioritize conducting more prospective studies over retrospective ones to demonstrate the efficacy and motivate the broader adoption of this technology. They also should conduct more long-term research to investigate if more precise targeting and optimal path planning will translate to improved clinical outcomes [50].

*Augmented Reality (AR) and Virtual Reality (VR)*—Another approach that should be further investigated is augmented reality-based interactive path planning, in which the neurosurgeons are provided with Holographic high-resolution 3D visualization of segmented anatomical structures [122]. In this approach, the neurosurgeon performs the path planning, but the software creates forbidden regions to protect the critical structures and prevent an unsafe trajectory from being planned. Furthermore, improving fusion technology to automatically fuse preoperative MR images, three-dimensional brain volume imaging, SWI, time-of-flight magnetic resonance angiography, and T1-weighted gadolinium-enhanced MRIs could improve the path-planning accuracy using AR-based path-planning approach [123]. In the future, more advanced VR-based path-planning simulators could be developed using current state-of-the-art technologies. These simulators would provide a platform to train neurosurgeons to manually generate more optimal and safer paths with greater efficiency or to more precisely evaluate paths generated by automatic path planners.

*Artificial Intelligence and Machine Learning*—With the rapid advancement of artificial intelligence in recent years, modern AI tools could play a crucial role in assisting neurosurgeons with path planning and decision-making. For instance, in multi-objective cost function-based path planning, AI tools could assist with weight tuning based on patient-specific recommendations from neurosurgeons or by training on data from previous patients. A notable example of such a system is AtlasGPT [124], which was recently released. This system can assist neurosurgeons in various aspects when trained on large datasets. Further developments in this research field could streamline the path-planning process, open new possibilities, and potentially lead to faster clinical adoption.

## 6. Conclusions

In conclusion, our systematic review encompasses the latest advancements in path-planning techniques for minimally invasive neurosurgical procedures, focusing on various tools like straight needles, steerable needles, and concentric tube robots. We have thoroughly analyzed a range of path-planning algorithms applicable to both single and multi-target scenarios, as well as the utilization of different imaging modalities and critical structures considerations during planning. Investigating multi-trajectory-planning methods for steerable needle and concentric tube robots failed to capture substantial attention and requires more investigation in the future. While recent progress in the field is promising, the wide adoption of automatic path-planning software requires further investigation into its safety and efficacy enhancing clinical outcomes.

## Figures and Tables

**Table 1:** Comparison of several relevant review papers published during the last couple of years.

	Neurosurgical Application	Various Types of Path-Planning Techniques	Papers Published after 2019	Straight Tool	Streeable Needle	Concentric Tube Robot
Starup-Hansen et al. [9]	Yes	No	Yes	Yes	No	No
Zanello et al. [10]	Yes	Yes	No	Yes	No	No
Ye et al. [11]	No	Yes	Yes	No	Yes	No
Our review paper	Yes	Yes	Yes	Yes	Yes	Yes

## Data Availability

The original contributions presented in the study are included in the article, further inquiries can be directed to the corresponding authors.
